# Quantitative CT Metrics Associated with Variability in the Diffusion Capacity of the Lung of Post-COVID-19 Patients with Minimal Residual Lung Lesions

**DOI:** 10.3390/jimaging9080150

**Published:** 2023-07-26

**Authors:** Han Wen, Julio A. Huapaya, Shreya M. Kanth, Junfeng Sun, Brianna P. Matthew, Simone C. Lee, Michael Do, Marcus Y. Chen, Ashkan A. Malayeri, Anthony F. Suffredini

**Affiliations:** 1 National Heart, Lung, and Blood Institute, National Institutes of Health, Bethesda, MD 20892, USA; 2 Critical Care Medicine Department, Clinical Center, National Institutes of Health, Bethesda, MD 20892, USA; 3 Radiology & Imaging Sciences Department, Clinical Center, National Institutes of Health, Bethesda, MD 20892, USA

**Keywords:** CT pulmonary vascular morphology, parenchymal radiodensity, alveolar volume, COVID-19, diffusion capacity of the lung

## Abstract

(1) Background: A reduction in the diffusion capacity of the lung for carbon monoxide is a prevalent longer-term consequence of COVID-19 infection. In patients who have zero or minimal residual radiological abnormalities in the lungs, it has been debated whether the cause was mainly due to a reduced alveolar volume or involved diffuse interstitial or vascular abnormalities. (2) Methods: We performed a cross-sectional study of 45 patients with either zero or minimal residual lesions in the lungs (total volume < 7 cc) at two months to one year post COVID-19 infection. There was considerable variability in the diffusion capacity of the lung for carbon monoxide, with 27% of the patients at less than 80% of the predicted reference. We investigated a set of independent variables that may affect the diffusion capacity of the lung, including demographic, pulmonary physiology and CT (computed tomography)-derived variables of vascular volume, parenchymal density and residual lesion volume. (3) Results: The leading three variables that contributed to the variability in the diffusion capacity of the lung for carbon monoxide were the alveolar volume, determined via pulmonary function tests, the blood vessel volume fraction, determined via CT, and the parenchymal radiodensity, also determined via CT. These factors explained 49% of the variance of the diffusion capacity, with *p* values of 0.031, 0.005 and 0.018, respectively, after adjusting for confounders. A multiple-regression model combining these three variables fit the measured values of the diffusion capacity, with R = 0.70 and *p* < 0.001. (4) Conclusions: The results are consistent with the notion that in some post-COVID-19 patients, after their pulmonary lesions resolve, diffuse changes in the vascular and parenchymal structures, in addition to a low alveolar volume, could be contributors to a lingering low diffusion capacity.

## 1. Introduction

A persistent reduction in the diffusion capacity of the lung for carbon monoxide (DLCO) is a frequently reported longer-term consequence of COVID-19 infection, with a cited prevalence of 40–65% in patients who have recovered from the disease [1,2,3,4,5,6,7,8,9,10,11]. Although the reduction in DLCO is correlated with the level of radiological anomalies in the lungs in the acute phase [12] and persisting abnormalities in the recovery phase [3,4,5,6,7,10], it has not been fully explained in patients who recovered with minimal residual radiologic abnormalities in the lungs. Previous studies have debated whether a lingering low DLCO after recovery is mainly due to reduced alveolar volume (VA) and not interstitial or vascular abnormalities [13] or involves the presence of abnormal perfusion or gas exchange [11,14].

The previous literature on chronic obstructive pulmonary disease suggests that image-based measures of diffuse changes in pulmonary vascular volume and morphology predict disease severity [15], and measures of parenchymal density reflect interstitial structural changes arising from emphysema or inflammation [16]. From this perspective, we performed a cross-sectional study of a group of post-COVID-19 patients with minimal residual abnormalities on radiologic exams. We studied a set of variables that may be associated with the variability of the diffusion capacity of the lung in these patients. In particular, we were confident in the conclusions of minimal residual lesions reached by our radiologists, some of whom have over 20 years of experience. Therefore, we were interested in quantitative CT (computed tomography) measurements of global characteristics that would not manifest as visible local abnormalities such as reticulations, nodules and bands [10]. Specifically, we obtained fully automated global measurements of vascular structure and parenchymal radiodensity from non-contrast high-resolution computed tomography (HRCT) scans of the lungs. To realize full automation without the potential bias of human operator input, we developed adaptive methods to account for the variabilities across different patients and CT scanner performances.

## 2. Materials and Methods

### 2.1. Study Population, Demographics and Pulmonary Function Tests

**Figure 1:**
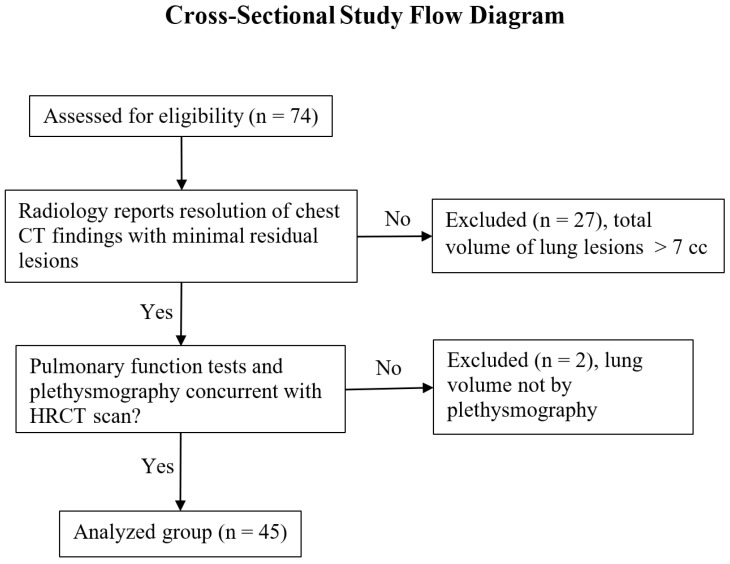
Flow chart of study inclusion.

We performed a retrospective cross-sectional study of participants enrolled in the clinical study “Cardiopulmonary inflammation and multi-system imaging during the clinical course of COVID-19 infection in asymptomatic and symptomatic persons” (clinicaltrials.gov NCT04401449) from December 2020 to April 2022 at the Clinical Center of the National Institutes of Health, USA. The study was approved by the National Institutes of Health IRB (IRB # 20CC0113). The participant inclusion criteria for this study were as follows: 1) adult patients who were enrolled in the clinical trial cited above; 2) those who underwent pulmonary function and plethysmography lung volume tests concurrent with a non-contrast HRCT chest scan in the recovery and/or convalescent phases of the disease from 4 weeks to a year after the onset of symptoms; and 3) radiology reports described the resolution of chest CT findings with zero or minimal residual lesions remaining, with the total volume of lesions less than 7 cc. The flow chart for study inclusion is illustrated in Figure 1.

Referring to Table 1, a total of 45 patients were analyzed in the study, including 23 male and 22 female patients, with an average age (1st quartile, 3rd quartile) of 45 (37, 57). Thirteen of the forty-five patients were vaccinated prior to COVID-19 infection. In terms of the severity of the disease in the acute phase, 34 were mild, 19 were moderate and 2 were severe. This was according to the National Institute of Allergy and Infectious Diseases ordinal scale of 1 to 8 (22), on which grades 1–2 are categorized as mild, grades 3–5 are categorized as moderate and grades 6 and above are categorized as severe.

The pulmonary function tests included spirometry, the diffusion capacity of the lung for carbon monoxide and lung volume measurements via plethysmography. The measures of diffusion capacity included the diffusion capacity of the lung for carbon monoxide adjusted for hemoglobin as a percentage of the predicted reference values [17], abbreviated as DLCO_adj (% predicted), and the corresponding transfer coefficient of DLCO_adj/alveolar volume as % of predicted, abbreviated as KCO_adj (% predicted) [17]. The diffusion capacity test also provided the measurement of the total alveolar volume as the % of the predicted value, or VA (% predicted).

### 2.2. Computed Tomography Imaging Analysis

**Figure 2:**
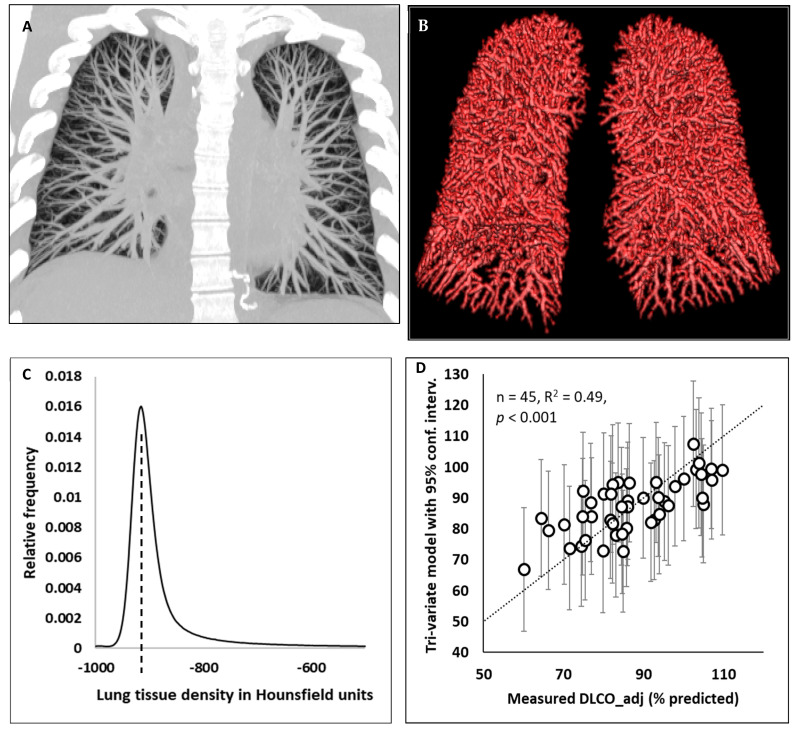
Illustrations of computed tomographic measurements and a tri-variate model of the diffusion capacity of the lung. (**A**) Maximum intensity projection of an HRCT scan of a post-COVID-19 patient, highlighting intraparenchymal arteries and veins. (**B**) The 3D structure of pulmonary blood vessels that were segmented from the HRCT images. (**C**) A histogram of the lung tissue radiodensity values of a patient in Hounsfield units. The mode (peak) of the histogram is marked by the dashed line. It represents the typical radiodensity of non-vessel parenchymal tissue. (**D**) The measured values of the DLCO_adj (% predicted) versus a tri-variate model combining the three variables that were statistically significantly associated with the DLCO_adj (% predicted). Error bars represent the 95% confidence intervals of the model values. The three variables are the alveolar volume (% predicted), blood vessel volume fraction, determined via CT, and the mode of the parenchymal radiodensity histogram, also determined via CT. The dotted line is the identity line.

The computed tomographic exams included a non-contrast lung HRCT protocol provided by the scanner manufacturer (Siemens Medical Solutions USA, Inc., Malvern, PA, USA). The scan yielded contiguous transverse sections covering the entire length of the chest (Figure 2A). The total volume of the residual lesions was measured manually according to findings in the routine radiological reports. Additionally, an automated software was developed to produce several image-based structural measurements, including the total intraparenchymal blood vessel volume (TBV), the blood vessel volume fraction, defined as the TBV/total non-vessel air-free lung tissue volume (TBV/TissueV) [15], the total non-vessel lung tissue mass (TissueM), and the mode of the histogram of the parenchymal radiodensity values (PDm) [16].

The vascular measurement procedure consisted of several steps. These include segmenting the lung volume [18], followed by calculating the total lung mass, for which the CT radiodensity values were converted into mass densities [19]; segmenting the intraparenchymal blood vessels in the images using a custom algorithm (IDL, L3Harris, Broomfield, CO, USA) (Figure 2B and Supplementary Video S1); calculating the total blood vessel volume (TBV), accounting for resolution-related partial volume effects; calculating the total non-vessel pulmonary tissue mass (TissueM) and air-free tissue volume (TissueV) [18,19]; and lastly, determining the blood vessel volume fraction as the ratio of TBV/TissueV [15]. A detailed description of the method is provided in Appendix A.

A novel feature of the vascular measurement was developed to overcome the so-called “partial volume effect” of CT scans. This effect describes the fact that in HRCT scans, blood vessels that are smaller than the resolution of the scanner may still be visible due to the higher radiodensity (X-ray attenuation) of blood compared to the surrounding aerated parenchyma tissue. However, these small vessels appear to be less dense (less bright) than bulk blood and tissue, such as the heart and the interiors of large vessels. This appearance comes from the fact that an image voxel may be partly occupied by a visible but small blood vessel, and the rest may be occupied by parenchymal tissue of lower density. Because of this effect, a direct summation of all voxels containing visible blood vessels would overestimate the total blood vessel volume, as evidenced by the appearance of “thickened” small vessels in Figure 1B. To correct for this effect, we developed an algorithm to calculate the blood vessel volume fraction for each voxel underlying a visible blood vessel, which is described in detail in Appendix A. The key element of the vascular measurement is a custom algorithm to determine the local radiodensity of non-vessel parenchyma as the basis for segmenting and calculating the blood vessel volume.

We also measured the mode of the histogram of the parenchymal radiodensity values in Hounsfield units throughout the lungs [20,21] (Figure 2C). This measurement is independent of the vascular measurements. It represents the typical radiodensity of the parenchymal tissue that includes the alveoli and the surrounding capillaries, small airways and extracellular matrix but excludes visible structures on the CT scan such as larger airways, blood vessels and the interlobular septa. The density histogram was adjusted to the state of maximal inspiration at total lung capacity to be compatible with the condition of laboratory-based diffusion measurements (see Appendix A for a detailed description). The mode of the radiodensity histogram is robust against dependent atelectasis that may occur during the CT scan [22].

### 2.3. Statistical Analysis

We determined the univariate Pearson correlation between the outcome measurement of DLCO_adj (% predicted) and a set of prespecified input variables. The input variables included the alveolar volume VA (% predicted), the CT-derived measurements of TBV, blood vessel volume fraction, TissueM, PDm and the total volume of residual lung lesions, the demographic variables of age, sex and vaccination status, the initial severity of the disease and the time between the onset of symptoms and the CT date. *p* values below 0.05 were regarded as statistically significant.

For the multi-variate analysis, we used a partial correlation analysis [23] to determine the independent contribution of each of the input variables to the variance in the outcome DLCO_adj (% predicted). We selected the three leading variables and used a multiple linear regression analysis to determine the statistical significance of their association with the outcome after adjusting for potential confounders. The confounders included all demographic variables and the volume of residual lung lesions on the CT scan. We then combined the three leading variables in a tri-variate linear regression model of DLCO_adj (% predicted) and calculated the 95% confidence interval of the modeled value for each subject. The tri-variate model was evaluated against the measured values of DLCO_adj (% predicted) for the entire cohort.

## 3. Results

The results of the pulmonary function and lung volume tests for all subjects are summarized in Table 1. Of the 45 subjects, 12 had a DLCO_adj (% predicted) value below 80%, one had a KCO_adj (% predicted) value below 80%, 4 had an FEV1 (% predicted) value below 80% and none had an FEV1/FVC (% predicted) value below 80%. Of the 45 subjects, 11 had a VA (% predicted) value below 80%. From the radiology reports, 25 of the 45 subjects had residual stable lesions including sub-centimeter nodules (calcified and non-calcified), scarring or atelectasis. The total volume of the residual lesions was between 0 and 7 cc.

The results of the univariate analysis between measures of the diffusion capacity of the lung and all input variables are summarized in Table 2. In terms of statistically significant associations (*p* < 0.05), DLCO_adj (% predicted) was positively correlated with the alveolar volume, the CT-derived total blood vessel volume (TBV) and the total non-vessel pulmonary tissue mass (TissueM). The transfer coefficient KCO_adj (% predicted) was negatively correlated with the alveolar volume and positively correlated with the CT-derived blood vessel volume fraction and the parenchymal radiodensity (PDm).

The results of the multivariate analysis are summarized in Table 3. From the partial correlation analysis, the three leading variables in terms of their independent contributions to the variance in DLCO_adj (% predicted) were the alveolar volume, the blood vessel volume fraction and the parenchymal radiodensity. These accounted for 27%, 10% and 12% of the variance in DLCO_adj (% predicted), respectively. They were followed by a distant fourth variable, the time between the onset of symptoms and the CT date, which accounted for 2.6% of the variance. All other variables accounted for less than 2% of the variance. In the multiple linear regression model, the three leading variables were significantly associated with DLCO_adj (% predicted) after adjusting for potential confounding variables, including the demographic variables and the volume of residual lung lesions (*p* = 0.031, 0.005 and 0.018, respectively). The other variables did not reach statistical significance (*p* > 0.17). A comparison of the measured values of DLCO_adj (% predicted) and the tri-variate model combining the three leading variables is shown in Figure 2D. The model explained 49% of the variance of the measured values, with *p* < 0.001.

## 4. Discussion

We performed a cross-sectional study of the pulmonary function results and CT scans of a group of patients at two months to a year after recovery from COVID-19 infection, when the radiologic abnormalities in the lungs had resolved with minimal residual lesions. We found that their spirometry results for FEV1 and FEV1/FVC had largely returned to normal levels, with median values of 95% and 96% of the predicted references. In contrast, their diffusion capacities of the lung for carbon monoxide and their alveolar volumes were both lower, with median values of 86% of the predicted references. Among this group, 27% (12 of 45) had DLCO_adj values below 80% of the predicted reference, while 9% (4 of 45) had FEV1 values below 80% of the predicted reference, and none had FEV1/FVC values below 80% of the predicted reference.

Although these pulmonary function and lung volume metrics on their own appear to indicate that the reduction in the diffusion capacity of the lung may have been caused mainly by a reduction in the alveolar volume, further analysis showed that the diffusion capacity of the lung was also associated with the vascular volume fraction of the lung tissue and the parenchymal density. These structural factors independently explained 22% of the variability in the DLCO_adj (% predicted) values among the subjects, in addition to the 27% that was explained by the alveolar volume. A multivariate model combining these factors explained about half of the variability in the diffusion capacity of the lung in this cohort.

The results are consistent with the notion that in post-COVID-19 patients with minimal residual pulmonary lesions, a lingering reduction in the diffusion capacity of the lung could be a multi-faceted phenomenon. In addition to lower alveolar volumes, diffuse changes in the vascular and parenchymal structures could be contributors to the reduced diffusion capacity for carbon monoxide. A limitation of this study is that we could not determine with certainty whether the variabilities in the vascular volume fraction and parenchymal radiodensity among the cohort resulted from the COVID-19 infection or were part of pre-existing backgrounds since few patients had baseline CT scans prior to contracting the disease.

The results overall suggest that in patients who had recovered from COVID-19 but had poor recovery of the diffusion capacity of the lung without clear correlates of radiologic abnormalities, it may be beneficial to assess certain global measures of vascular and parenchymal structures in the lungs from HRCT scans as potential contributors to low DLCO.

## Figures and Tables

**Table 1:** Patient demographics and pulmonary function results presented as medians (1st quartile, 3rd quartile). Definition of abbreviations: DLCO_adj—diffusion capacity of the lung for carbon monoxide, adjusted for hemoglobin; KCO_adj—carbon monoxide transfer coefficient, adjusted for hemoglobin, DLCO_adj/alveolar volume; FEV1—forced expiratory volume in the first second; FEV1/FVC—ratio of forced expiratory volume in the first second over forced vital capacity; VA—alveolar volume; CT—computed tomography.

Variables	All Patients (n = 45)
Age, year	45 (37, 57)
Sex	23 male and 22 female
Vaccination status	13 vaccinated and 32 unvaccinated
Disease severity in the acute phase	34 mild, 9 moderate and 2 severe
Days between symptom onset and CT scan	84 (76, 95)
Total volume of residual pulmonary lesions on CT (cc)	0.02 (0, 0.8)
DLCO_adj (% predicted)	86 (80, 96)
KCO_adj (% predicted)	102 (91, 111)
FEV_1_ (% predicted)	95 (84, 103)
FEV_1_/FVC (% predicted)	96 (92, 100)
VA (% predicted)	86 (80, 93)

**Table 2:** List of all input variables and their univariate Pearson correlation with the measures of the diffusion capacity of the lung (n = 45). The demographic variables and the total volume of residual lesions on CT were considered potential confounders in the multivariate analysis (Table 3). Definition of abbreviations: VA—alveolar volume; CT—computed tomography; TBV—total intraparenchymal blood vessel volume by CT; TissueM—total non-vessel lung mass by CT; PDm—mode of the histogram of parenchymal radiodensity values on CT.

Input Variables	DLCO_adj (% Predicted)	KCO_adj (% Predicted)
VA (% predicted)	r = 0.52, *p* < 0.001	r = −0.39, *p* = 0.008
TBV	r = 0.41, *p* = 0.005	r = 0.13, *p* = 0.39
Blood vessel volume fraction	r = 0.30, *p* = 0.046	r = 0.36, *p* = 0.014
TissueM	r = 0.29, *p* = 0.048	r = −0.08, *p* = 0.58
PDm	r = 0.21, *p* = 0.16	r = 0.33, *p* = 0.027
Total volume of residual lung lesions on CT	r = −0.21, *p* = 0.17	r = −0.29, *p* = 0.051
Age	r = −0.11, *p* = 0.47	r = −0.05, *p* = 0.73
Sex	r = 0.02, *p* = 0.88	r = 0.14, *p* = 0.34
Vaccination status	r = 0.13, *p* = 0.40	r = 0.02, *p* = 0.91
Disease severity in the acute phase	r = −0.08, *p* = 0.62	r = −0.01, *p* = 0.94
Days between symptom onset and CT date	r = −0.07, *p* = 0.63	r = −0.29, *p* = 0.051

**Table 3:** Summary of multivariate analysis for the outcome measurement of DLCO_adj (% predicted). Among all the input variables listed in Table 2, the leading three variables by their independent contributions to the variance in DLCO_adj were the alveolar volume (% predicted), the blood vessel volume fraction and the parenchymal radiodensity. The statistical significances of their associations with DLCO_adj after adjusting for potential confounders were provided by the results of the multiple linear regression model. The tri-variate model of DLCO_adj (% predicted) combines the leading three input variables. Definition of abbreviations: VA (% pred.)—alveolar volume, % of predicted reference; Blood vessel V.F.—blood vessel volume fraction; PDm—mode of parenchymal radiodensity histogram measured by CT.

	VA (% Pred.)	Blood Vessel V.F.	PDm	Days Since Symptom Onset	All Other Input Variables	Tri-Variate Model
Independent contribution to the variance of DLCO_adj (% pred.), n = 45	27%	10%	12.3%	2.6%	<2%	49.4%
Multiple linear regression model beta coefficient and *p* value, n = 45	β = 0.66, *p* = 0.031	β = 186, *p* = 0.005	β = 0.27, *p* = 0.018	β = −0.03, *p* = 0.17	*p* > 0.25	*p* < 0.001

## Data Availability

Data underlying graphs are available at https://nhlbi.figshare.com/ (accessed on 25 July 2023).

## Supplementary Materials

The following supporting information can be downloaded at: https://www.mdpi.com/article/10.3390/jimaging9080150/s1, Video S1: Three-dimensional structure of pulmonary blood vessels in a post COVID-19 patient.

## Appendix A

*Automated procedure of obtaining vascular measurements*: In non-contrast HRCT images of the lung, intraparenchymal blood vessels smaller than the scanner resolution may still be visibly brighter than the surrounding aerated parenchymal tissue due to the higher density and X-ray attenuation of blood. However, small vessels, as well as the edges of larger vessels, appear less bright than bulk blood such as the chambers of the heart and the interiors of large vessels. This is due to the fact that an image voxel is partly occupied by a small blood vessel or by the border of a larger blood vessel while the rest of the voxel is occupied by aerated parenchymal tissue. Because of this so-called partial volume effect, a direct summation of all image voxels that represent blood vessels will overestimate the total volume of the blood vessels. With this understanding, our procedure for vascular measurements consisted of the following steps: Step 1: Segmenting the lung volume based on a threshold of radiodensity values in Hounsfield units (HU), which allows for the calculation of a total lung mass by converting the radiodensity values in HU into mass density values in g/mL. Step 2: Determining the local radiodensity of non-vessel parenchyma for every image voxel in the lungs using a custom local histogram algorithm (IDL, L3Harris, Broomfield, CO, USA), followed by identifying all voxels with radiodensity values above a threshold relative to the local parenchyma and further identifying a subset of voxels with a continuous path to the heart. These voxels are recognized as underlying visible blood vessels. Step 3: Calculating the blood vessel volume fraction in each of the voxels identified in Step 2 using the following formula:

Blood vessel volume fraction = [I(x,y,z) − Iparenchyma(x,y,z)]/[Iblood − Iparechyma(x,y,z)],

where I(x,y,z) is the radiodensity of the voxel located at the coordinates (x, y, z), Iblood is the radiodensity of the blood pool and Iparenchyma(x, y, z) is the radiodensity of the local parenchyma. Step 4. Summing the product of (vessel volume fraction*voxel volume) over all voxels identified in the previous step to produce the measurement of the total blood vessel volume (TBV). Step 5: Calculating the total non-vessel tissue mass and tissue volume in the lungs using the following formulas:

total non-vessel lung mass = total lung mass – TBV × (Iblood + 1000)/1000, total non-vessel tissue volume (TissueV) = total non-vessel lung mass/(1.065 g/mL),

where the value 1.065 g/mL is the mass density of the gas-free parenchymal tissue [24]. Lastly, we obtained the measurement of the overall blood vessel volume fraction in the lung tissue as the ratio of TBV/TissueV.

*Adjusting the radiodensity histogram to the state of maximal inspiration*: We first determined from the HRCT the frequency histogram of the radiodensity values in Hounsfield units throughout the lungs, herein denoted as H(ρ), where ρ is the radiodensity. We then transformed the histogram to the state of maximal inspiration to the total lung capacity (TLC) using the following formula of mass conservation:

H’(ρ) = H(ρ)/[(1 − ρ/1.065)Vair/TLC + ρ/1.065],

where H’(ρ) is the projected histogram at the state of maximal inspiration, Vair is the total air volume in the lungs, as measured from the HRCT images, and the value 1.065 g/mL is the mass density of the gas-free parenchymal tissue [24].
